# Genome Report: chromosome-scale genome assembly of the African spiny mouse (*Acomys cahirinus*)

**DOI:** 10.1093/g3journal/jkad177

**Published:** 2023-08-08

**Authors:** Elizabeth Dong Nguyen, Vahid Nikoonejad Fard, Bernard Y Kim, Sarah Collins, Miranda Galey, Branden R Nelson, Paul Wakenight, Simone M Gable, Aaron McKenna, Theo K Bammler, Jim MacDonald, Daryl M Okamura, Jay Shendure, David R Beier, Jan Marino Ramirez, Mark W Majesky, Kathleen J Millen, Marc Tollis, Danny E Miller

**Affiliations:** Department of Pediatrics, University of Washington, Seattle, WA 98195, USA; Center for Developmental Biology & Regenerative Medicine, Seattle Children's Research Institute, Seattle, WA 98101, USA; Brotman Baty Institute for Precision Medicine, University of Washington, Seattle, WA 98195, USA; School of Informatics, Computing, and Cyber Systems, Northern Arizona University, Flagstaff, AZ 86011, USA; Department of Biology, Stanford University, Stanford, CA 94305, USA; Center for Developmental Biology & Regenerative Medicine, Seattle Children's Research Institute, Seattle, WA 98101, USA; Division of Genetic Medicine, Department of Pediatrics, University of Washington, Seattle, WA 98195, USA; Center for Integrative Brain Research, Seattle Children's Research Institute, Seattle, WA 98101, USA; Center for Integrative Brain Research, Seattle Children's Research Institute, Seattle, WA 98101, USA; School of Informatics, Computing, and Cyber Systems, Northern Arizona University, Flagstaff, AZ 86011, USA; Department of Molecular & Systems Biology, Dartmouth Geisel School of Medicine, Lebanon, NH 03755, USA; Department of Environmental & Occupational Health Sciences, University of Washington, Seattle, WA 98195, USA; Department of Environmental & Occupational Health Sciences, University of Washington, Seattle, WA 98195, USA; Department of Pediatrics, University of Washington, Seattle, WA 98195, USA; Center for Developmental Biology & Regenerative Medicine, Seattle Children's Research Institute, Seattle, WA 98101, USA; Brotman Baty Institute for Precision Medicine, University of Washington, Seattle, WA 98195, USA; Department of Genome Sciences, University of Washington, Seattle, WA 98195, USA; Allen Discovery Center for Cell Lineage Tracing, Seattle, WA 98195, USA; Howard Hughes Medical Institute, Seattle, WA 98195, USA; Institute of Stem Cell & Regenerative Medicine, University of Washington, Seattle, WA 98195, USA; Department of Pediatrics, University of Washington, Seattle, WA 98195, USA; Center for Developmental Biology & Regenerative Medicine, Seattle Children's Research Institute, Seattle, WA 98101, USA; Center for Integrative Brain Research, Seattle Children's Research Institute, Seattle, WA 98101, USA; Department of Neurological Surgery, University of Washington, Seattle, WA 98195, USA; Department of Pediatrics, University of Washington, Seattle, WA 98195, USA; Center for Developmental Biology & Regenerative Medicine, Seattle Children's Research Institute, Seattle, WA 98101, USA; Institute of Stem Cell & Regenerative Medicine, University of Washington, Seattle, WA 98195, USA; Department of Laboratory Medicine & Pathology, University of Washington, Seattle, WA 98195, USA; Department of Pediatrics, University of Washington, Seattle, WA 98195, USA; Brotman Baty Institute for Precision Medicine, University of Washington, Seattle, WA 98195, USA; Center for Integrative Brain Research, Seattle Children's Research Institute, Seattle, WA 98101, USA; School of Informatics, Computing, and Cyber Systems, Northern Arizona University, Flagstaff, AZ 86011, USA; Brotman Baty Institute for Precision Medicine, University of Washington, Seattle, WA 98195, USA; Division of Genetic Medicine, Department of Pediatrics, University of Washington, Seattle, WA 98195, USA; Department of Laboratory Medicine & Pathology, University of Washington, Seattle, WA 98195, USA

**Keywords:** genome assembly, *Acomys cahirinus*, spiny mouse, regenerative wound healing, Nanopore sequencing

## Abstract

There is increasing interest in the African spiny mouse (*Acomys cahirinus*) as a model organism because of its ability for regeneration of tissue after injury in skin, muscle, and internal organs such as the kidneys. A high-quality reference genome is needed to better understand these regenerative properties at the molecular level. Here, we present an improved reference genome for *A. cahirinus* generated from long Nanopore sequencing reads. We confirm the quality of our annotations using RNA sequencing data from 4 different tissues. Our genome is of higher contiguity and quality than previously reported genomes from this species and will facilitate ongoing efforts to better understand the regenerative properties of this organism.

## Teaser

This work presents an improved reference genome for the African spiny mouse *Acomys cahirinus*, a promising model organism known for its remarkable tissue regeneration abilities. Nanopore long-read sequencing was used to improve the genome's contiguity and quality, and RNA sequencing data from various tissues support the accuracy of the annotations. This high-quality genome will aid in advancing our understanding of the molecular mechanisms underlying *A. cahirinus*’ regenerative properties.

## Introduction

African spiny mice (genus *Acomys*) are a rodent species native to Africa and the Middle East. Their origin dates back to the late Miocene period ∼8.7 MYA in the savannas of East Africa (Aghová *et al.* 2019). Unique adaptations to their environment have made them distinct from other rodents, as they are the first rodent species to exhibit menstruation (Bellofiore *et al.* 2017, 2021) and have the unique ability to concentrate urine to survive their arid environments (Dickinson *et al.* 2007). The African spiny mouse inhabits what is known as Evolution Canyon in lower Nahal Oren, Mount Carmel, Israel which consists of 2 distinct microenvironments, the hot and dry African slope and the temperate, humid, and forest European slope (Hadid *et al.* 2014). The spiny mouse has thus been an evolutionary model of sympatric speciation, with populations of animals demonstrating divergence in karyotype (Volobouev *et al.* 2007), mitochondrial DNA (Hadid *et al.* 2014), and genome methylation patterns (Wang *et al.* 2022).

More recently, *Acomys cahirinus* (Desmarest, 1819), a member of the African spiny mouse family, has emerged as a model organism for the study of organ regeneration. Members of this family have adapted for survival in unique ways, including the ability for scarless healing of complex tissue after injury as adults. Spiny mice can shed their dorsal skin to escape the grasp of predators and then fully regenerate the lost skin without fibrotic scarring (Seifert *et al.* 2012). This scarless healing is accompanied by complete regeneration of skin including hair follicles, sebaceous glands, cartilage, adipose tissue, nerves, and blood vessels in the correct architecture for restoration of structure and function of skin tissue (Seifert *et al.* 2012; Brant *et al.* 2015; Gawriluk *et al.* 2016; Matias Santos *et al.* 2016; Jiang *et al.* 2019; Maden and Brant 2019; Brewer *et al.* 2021; Harn *et al.* 2021). The spiny mouse also demonstrates the ability to restore skeletal muscle after damage induced by cardiotoxin (Garry *et al.* 2016; Maden *et al.* 2018). These healing properties extend to internal organs; kidney damage induced using aggressive models of obstructive and ischemic injury is followed by complete regeneration of functional kidney tissue without scarring (Okamura *et al.* 2021). The spiny mouse has also been shown to exhibit resistance to myocardial ischemia and minimal scarring, as well as improvement in cardiac function after injury (Koopmans *et al.* 2021; Peng *et al.* 2021; Qi *et al.* 2021). Regeneration to this degree has been demonstrated in other mammalian species (albeit rarely), including humans, particularly in fetal tissues (Colwell *et al.* 2003; Drenckhahn *et al.* 2008; Porrello *et al.* 2011; Pratsinis *et al.* 2019; Abrams *et al.* 2021). This suggests that the potential pathways directing regeneration exist in the mammalian genome in a repressed state. A deeper understanding of the spiny mouse genome would help uncover its wound healing properties and possible reversal in nonregenerative mammalian species.

Here, we report a long-read-based chromosome-level assembly for the African spiny mouse *A. cahirinus*, a member of the *Acomys* family that is known to be capable of organ regeneration (Brewer *et al.* 2021; Okamura *et al.* 2021). We found that the genome of *A. cahirinus* is 2.3 Gb in length and contains >40% repetitive DNA. While previously published reference genomes for the species (Wang *et al.* 2022) contained a reported 94% gene completeness and 108-Mb scaffold contiguity, our assembled *A. cahirinus* genome is more contiguous, with a scaffold N50 of 127 Mb, as well as more complete in terms of gene content (98.5%). The *A. cahirinus* genomic resources provided here will contribute to the better understanding of their unique organismal adaptations broadly, while accelerating further discovery of mechanisms underlying their novel adult regenerative capabilities.

## Materials and methods

### Karyotype and banding

Chromosome analysis was performed on fibroblasts grown from ear tissue, anticoagulated blood, and bone marrow from the femur of a male *A. cahirinus*. Fibroblasts were grown to 70–80% confluency in DMEM/F12 with 10% fetal bovine serum (FBS) and 1% Pen-Strep, with rounded cells indicating active mitosis from passage 1, 2, or 3. Anticoagulated blood was grown in RPMI (Gibco #11875093) supplemented with 10% FBS and 1% Pen-Strep and 200 µL of PHA (Gibco #10576015) for 3 days. Femurs were cut open and rinsed multiple times with 1–2 mL of RPMI supplemented with 10% FBS and 1% Pen-Strep. Bone marrow cells were then placed into 10-mL cultures for 24 hours.

Samples were placed in 50 µL of ethidium bromide (1 mg/mL) and 50 µL of Karyomax Colcemid (10 µg/mL) (Gibco #15212-012) for 1 hour. Cells were then spun down at 500 g for 10 minutes. Cells were gently resuspended in 0.56% KCl and incubated at room temperature for 20 minutes. Cells spun down again at 500 g for 10 minutes. Cells were gently resuspended in Carnoys Fixitive (3:1 methanol:acetic acid) and incubated for 45 minutes. This was repeated twice, with incubation shortened to 10 minutes. Cells were then resuspended in a small volume of fresh Carnoys and dropped onto clean slides. Slides were kept at 37° for a minimum of 24 hours before banding.

For GTG banding, slides were dipped in Trypsin 2.5% (Gibco #15090-046) with NaCl for 15–60 seconds, then rinsed in NaCl with FBS, then NaCl again. Slides were then stained for 10 minutes in Karyomax Giemsa Stain R66 Solution (Gibco #10092-013) with 50 mL of Gurr Buffer Tablets 6.8ph (Gibco #10582-013). After rinse with ddH20, slides were dried and imaged.

### Nanopore sequencing and preassembly filtering

Genomic DNA was extracted from blood from a single male *A. cahirinus* animal using a Monarch HMW DNA Extraction Kit for Cells & Blood (T3050, New England Biolabs, Ipswich, MA) following the manufacturer's recommended protocol. DNA was quantified prior to library construction using the Qubit DNA HS Assay (ThermoFischer, Waltham, MA), and DNA fragment lengths were assessed using the Agilent Femto Pulse System (Santa Clara, CA). Libraries were prepared for sequencing using the Oxford Nanopore ligation kit (SQK-LSK110) following the manufacturers’ instructions, except that DNA repair and A-tailing were performed for 30 minutes and the ligation was allowed to continue for 1 hour. Prepared libraries were quantified using a Qubit fluorometer, and 30 fmol of the library was loaded onto a Nanopore version R.9.4.1 flow cell on the PromethION platform running MinKNOW version 21.05.20. To increase output, the flow cell was washed after approximately 24 hours of sequencing then an additional 12 fmol of library was loaded and run for an additional 48 hours. Basecalling was performed using Guppy 5.0.12 (Oxford Nanopore) using the super accuracy model (dna_r9.4.1_450bps_sup_prom.cfg). Reads of quality 6 or less were discarded, and NanoPlot was used to collect read statistics (Supplementary Table 1, Supplementary Fig. 1).

### Assembly and polishing

FASTQ files for assembly were extracted from unaligned bam files using samtools (Li *et al.* 2009) then Flye version 2.9 for assembly using the --nano-hq flag (Kolmogorov *et al.* 2019). Haplotigs and overlaps in the assembly were purged using purge_dups (Guan *et al.* 2020). The assembly was then polished using Medaka version 1.4.2 (https://github.com/nanoporetech/medaka) followed by a second polishing step with pilon version 1.24 (Walker *et al.* 2014). Assembly statistics at each step were generated using Quast (Gurevich *et al.* 2013) and BUSCO version 5.2.2 using the vertebrata_odb10 database (Simão *et al.* 2015) (Supplementary Table 2).

### Hi-C scaffolding

The primary contigs assembled from the Nanopore data were anchored to pseudo-chromosomes using 505,210,505 read pairs of a Hi-C library isolated from another *A. cahirinus* individual of unknown sex, downloaded from the NCBI Short Read Archive (SRX13258644) (Wang *et al.* 2022). After aligning the Hi-C reads with the ArimaHi-C Mapping Pipeline (https://github.com/ArimaGenomics/mapping_pipeline), YaHS v1.0 (Zhou *et al.* 2023) was used with default error correction for scaffolding, and Juicebox v1.11.08 (Dudchenko *et al.* 2018) was used to generate a Hi-C contact map.

### Annotation

Progressive Cactus was used (Armstrong *et al.* 2020) to perform a whole-genome alignment of the scaffolded *A. cahirinus* draft assembly to the *Mus musculus* GRCm39 reference genome (RefSeq GCF_000001635.27_GRCm39). Comparative annotation of the draft genomes was then performed using the Comparative Annotation Toolkit (CAT) (Fiddes *et al.* 2018). Briefly, the *M. musculus* RefSeq annotation GFF was parsed and validated with the “parse_ncbi_gff3” and “validate_gff3” programs (respectively) from CAT. The *M. musculus* reference transcript cDNA sequences were downloaded and mapped to the *M. musculus* draft genome with minimap2 (Li 2018) and provided to CAT as long-read RNA-seq reads in the “[ISO_SEQ_BAM]” field of the configuration file. For *A. cahirinus*, bulk RNA-seq data obtained from multiple pooled organs were downloaded from NCBI SRA BioProject PRJNA342864 (Bellofiore *et al.* 2017) and mapped to the draft assembly with STAR (Dobin *et al.* 2013) then provided to CAT in the “[BAMS]” field. CpG islands were identified using the cpg_lh utility from the UCSC suite of tools (Kent *et al.* 2002).

We modeled repeats de novo for the *A. cahirinus* scaffolds with RepeatModeler v2.0 (Flynn *et al.* 2020), and used RepeatMasker v4.1.3 (Smit *et al.* 1996) to (1) classify the de novo repeat family consensus sequences and (2) annotate all classified repeats in the genome assembly based on the “rodentia” repeat library from RepBase v4.0.7 (Bao *et al.* 2015).

### RNA isolation and mapping

Tissues (blood, heart, liver, and testis) were collected from an adult male *A. cahirinus* and homogenized, and RNA isolation, library generation, and sequencing were performed as previously described (Brewer *et al.* 2021; Okamura *et al.* 2021). Briefly, total RNA was extracted in Trizol solution (Ambion), DNase treated, and purified (PureLink RNA Mini Kit, Thermo Fisher Scientific). RNA was processed with KAPA's Stranded mRNA-Seq kit (Illumina) following the manufacturer's protocol in duplicate for each sample. The resulting libraries were assessed for library quality using fragment length and number of cycles in real-time PCR. Passing samples were sequenced on a NextSeq 500 using a 300-cycle mid-output kit, with paired 150-bp reads.

RNA was mapped to the final assembly using bwa (version 0.7.17-r1188) (Li and Durbin 2009). Reads mapping to genomic features defined in the GTF file were counted using featureCounts using the simplified file format (Liao *et al.* 2014). For each gene, the transcripts per million (TPM) value was calculated using only mapped reads (Supplementary File 2). Venn diagrams were created in R and show overlap in genes from each tissue with TPM values greater than 2 (Fig. 2b).

### Comparative genomics

#### Synteny analysis

To understand evolutionary change between the *A. cahirinus* and *M. musculus* genomes, we used SynMap2 (Haug-Baltzell *et al.* 2017) on the CoGe platform (Lyons and Freeling 2008) to visualize whole-genome synteny between *A. cahirinus* scaffolds and the *M. musculus* reference genome (mm39). We used lastz (Harris 2007) to map *A. cahirinus* coding sequences to both genomes, DAGChainer (Haas *et al.* 2004) to compute chains of syntenic genes, and CodeML (Yang and Nielsen 2002) to calculate the rate of nonsynonymous (Kn) and synonymous (Ks) substitutions, as well as their ratios (Kn/Ks), with default parameters.

#### Repeat analysis

To estimate the amount of evolutionary divergence within repeat families, we generated repeat family-specific alignments using the -a flag in RepeatMasker and calculated the average Kimura 2-parameter (K2P) sequence divergence between each annotated repeat insertion and its family consensus sequence. To correct for higher mutation rates at CpG sites, we weighted 2 transition mutations as 1% of a single transition. These steps were undertaken using the calcDivergenceFromAlign.pl tool in RepeatMasker. We compared the resulting repeat landscape obtained for *A. cahirinus* to a parallel analysis we conducted for *M. musculus* (mm10).

#### Orthologous gene analysis

To further examine genomic differences between *A. cahirinus* and *M. musculus*, we generated pairwise genome alignments. We first aligned *A. cahirinus* scaffolds as queries to the mouse reference genome (mm39) with lastz (Harris 2007) using parameters K = 2400, L = 3000, Y = 9400, H = 2000, which are sensitive enough to detect orthologous exons in placental mammals (Sharma and Hiller 2017), and a default scoring matrix, followed by chaining and netting (Kent *et al.* 2003). To analyze protein-coding genes, we downloaded mm39 RefSeq gene annotations for each mouse chromosome in whole gene BED format from the UCSC Genome Browser (Kent *et al.* 2002) and used the “stitch gene blocks” tool available on Galaxy (usegalaxy.org, last accessed February 2023) to reconstruct sequence alignments for each mouse protein-coding gene ID containing the prefix “NM_” (Blankenberg *et al.* 2011). We then removed gaps in the reference alignments, removed codons with missing nucleotides which produce unknown amino acids, removed premature stop codons, and converted all filtered FASTA alignments into axt format with AlignmentProcessor.py (https://tinyurl.com/23y38664, last accessed February 2023).

Finally, we used OrthoFinder v2.5.5 (Emms and Kelly 2019) to detect orthologs and identify gene duplication events in the evolutionary history of 12 therian mammalian genomes. In addition to *A. cahirinus*, we included the proteomes from the NCBI-annotated genome assemblies for opossum (*Monodelphis domestica*, GCA_027917375.1); African savannah elephant (*Loxodonta africana*, GCA_030077915.1); blue whale (*Balaenoptera musculus*, GCA_008658375.2); cow (*Bos taurus*, GCA_905123515.1); dog (*Canis lupus familiaris*, GCA_000002285.4), rhesus macaque (*Macaca mulatta*, GCA_003339765.3); human (*Homo sapiens*, GCA_000001405.29); guinea pig (*Cavia porcellus*, GCA_000151735.1); black rat (*Rattus rattus*, GCA_011064425.1); house mouse (mm10, above); and golden spiny mouse (*Acomys russatus*, GCA_903995435.1). All alignments and OrthoFinder output are included in Supplementary Data and are publicly available.

#### Functional analysis

To examine protein-coding differences that may point to selection pressures acting on genes since the divergence of *A. cahirinus* and *M. musculus*, we estimated the pairwise synonymous Ka and nonsynonymous Ks substitution rate, as well as the rate ratio Ka/Ks for all filtered axt gene alignments with KaKs_calculator2.0 (Wang *et al.* 2010), accounting for variable mutation rates across sites with a maximum likelihood method MS (Supplementary File 1). We concatenated the results of the Ka/Ks test for each gene ID and applied the false discovery rate (FDR = 0.05) to reduce false positives (Supplementary Data). We functionally annotated all unique gene IDs with Ka/Ks > 1.0 and an adjusted *P*-value < 0.05 using DAVID (Sherman *et al.* 2022) and Gene Ontology enrichment (Gene Ontology Consortium 2015), applying Benjamini–Hochberg and FDR corrections to adjust for multiple testing.

## Results and discussion

**Fig. 1.**
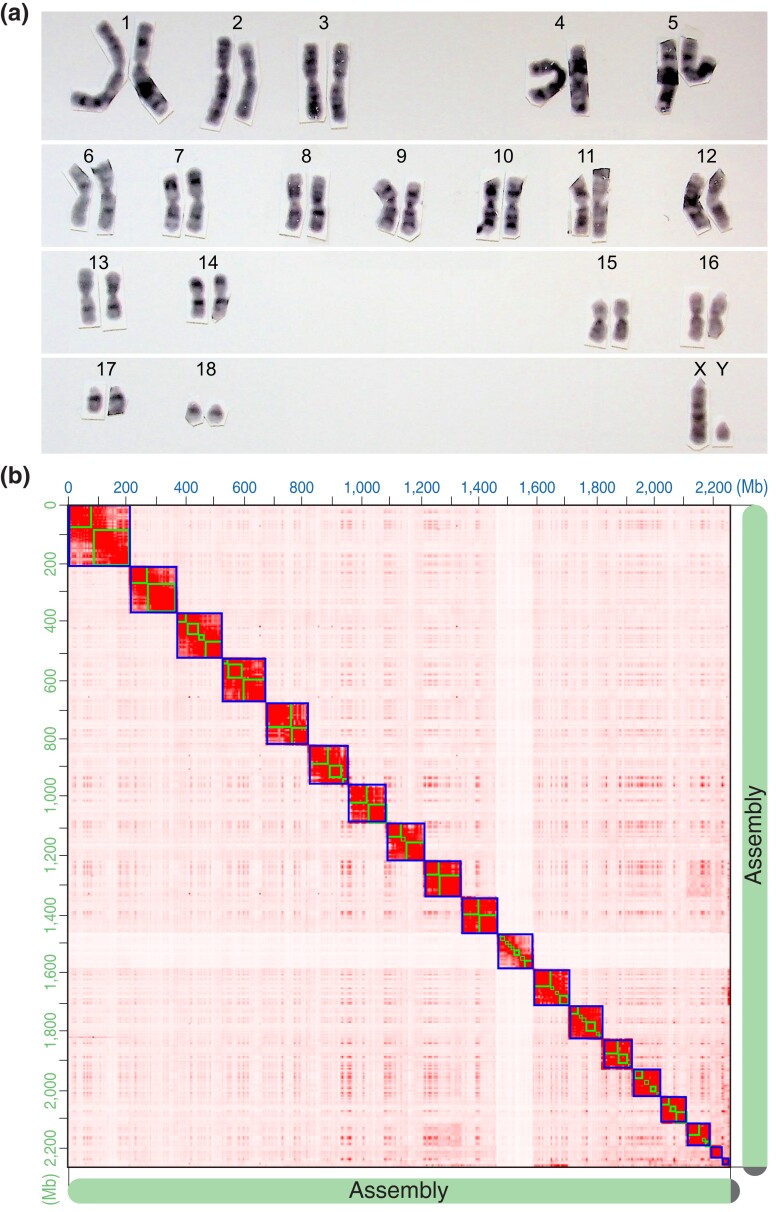
Karyotype of *Acomys cahirinus* and results of Hi-C scaffolding of the assembly. a) Representative karyotype of a male *A. cahirinus* from the same colony that DNA and RNA were obtained from for sequencing and assembly. Autosomes are arranged by size and numbered accordingly. b) Hi-C interaction contact heatmap of 19 *A. cahirinus* pseudo-chromosomes matches the karyotype (bin size is 1 Mb).

*A. cahirinus* from our colony have a chromosomal count of 38 (19 pairs). Most autosomes are metacentric or submetacentric with a large acrocentric X, small acrocentric Y, and 2 pairs of small acrocentric autosomes (Fig. 1a). The *A. cahirinus* karyotype, while similar in its combination of metacentric and acrocentric chromosomes to other rodent species, is karyotypically divergent from the completely acrocentric pairs of 20 chromosomes in *M. musculus*. The high karyotypic diversity in rodents enables chromosomal numbers and morphology to be a useful tool in species identification. Although the geographic origin of our animals is unknown, we find that our results match the *A. cahirinus* karyotype from Moreshet, Israel, which is distinct from the *A. cahirinus* karyotype generated from animals in Sinai, Egypt, which have 36 chromosomes (Volobouev *et al.* 2007).

Using a single male individual from our colony, we generated 87.5 Gb of Nanopore data for primary assembly with a read length N50 of 63 kb and a mean read quality of 13. The initial primary assembly after purging duplicates and polishing contained 181 contigs with a contig N50 of 58.8 Mb, a longest contig of 126.8 Mb, and a total length of 2.3 Gb (Table 1). The contigs were anchored to 19 pseudo-chromosomes based on the Hi-C scaffolding, matching expectations from the karyotype (Fig. 1b). Hi-C scaffolding reduced the number of assembled sequences to 129, with a scaffold N50 of 127 Mb and a total length of 2,289,268,912 bp and 79 gaps. All nanopore contigs were scaffolded. Fifty percent of the scaffolded assembly resides on 8 scaffolds (L50). According to the BUSCO analysis of the scaffolded assembly, 98.5% of complete and partial single-copy mammalian orthologs are present, indicating a higher level of completeness than previously published reference genomes for *A. cahirinus* (Wang *et al.* 2022)

**Table 1.** Comparison of metrics from available genome assemblies for *Acomys cahirinus*.

	Broad Institute (GCA_004027535.1)		Wang *et al.* (2022)		Current study	
	Contigs	Scaffolds	Contigs	Scaffolds	Contigs	Scaffolds
Total length	2.3 Gb	2.3 Gb	2.3 Gb	2.3 Gb	2.3 Gb	2.3 Gb
Number of sequences	391,811	371,342	120	108	181	129
N50	42.5 kb	65.4 kb	55.0 Mb	nr	58.8 Mb	127.8 Mb
L50	15,859	10,134	na	nr	16	8
Number of gaps (≥5 bp)	20,469		nr		79	
Complete + partial BUSCOs (Mammalia orthoDBv10)	83.1%		94%^*a*^		98.5%	

bp, base pairs; kb, kilobase pairs; Mb, megabase pairs; Gb, gigabase pairs; nr, not reported.

Unknown BUSCO database.

**Fig. 2.**
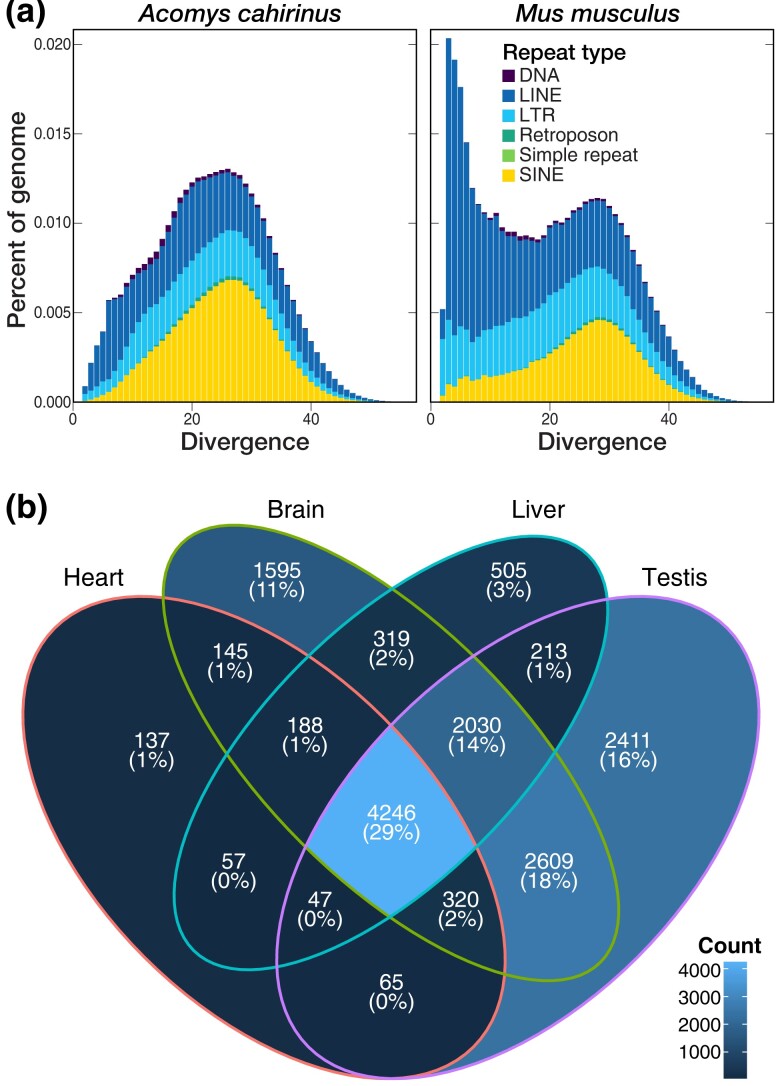
a) Repeat landscapes for *Acomys cahirinus* and *Mus musculus*, visualizing the percent of each genome comprised of different types of repeats according to their Kimura 2-parameter divergence from family consensus. DNA, DNA transposon; LINE, long interspersed nuclear element; LTR, long terminal repeat retrotransposon; SINE, short interspersed nuclear element. b) Venn diagram showing the number of genes per tissue with a TPM value greater than 2. The majority of genes (4,246; 29%) are expressed at this level in all 4 tissues, while testis had the highest number of genes (2,411; 16%) with a TPM greater than 2 not seen in other tissues.

We estimate that approximately 37% of the *A. cahirinus* genome consists of repetitive sequences, an identical proportion to what we found in *M. musculus* (Table 2). Thirty four percent of the *A. cahirinus* genome consisted of interspersed repeats such as transposable elements, with most of these belonging to retrotransposons, which accounted for 30% of the genome alone. Compared to *M. musculus*, *A. cahirinus* contains more SINE retrotransposons (8.3% of the genome vs 11.4%, respectively), while *M. musculus* contains more long interspersed nuclear elements (LINEs) (19% of the genome vs 11%, respectively). These differences can be attributed to a recent burst of LINE-1 retrotransposon activity in *M. musculus* (Sookdeo *et al.* 2013), as demonstrated by a relatively taller peak of LINE elements in the *M. musculus* genome at ≤10% K2P divergence compared to *A. cahirinus* (Fig. 2a). The pseudochromosome 11 (chr11), which is 4,798,714 bp in length and made of 11 scaffolds and 12 contigs, contained relatively fewer repeats (2,441) compared to other scaffolds.

**Table 2.** Genome annotation information for *Acomys cahirinus* (current study) and *Mus musculus* (mm10).

Parameters	Current study (*Acomys cahirinus*)	*Mus musculus* (mm10)
Total length	2.3 Gb	2.7 Gb
GC content	42.8%	41.7%
Annotated protein-coding genes	19,818	22,192
Average number of exons per gene	14.75	5.91
Average gene length	50,140 bp	28,506 bp
Average number of isoforms per gene	4.34	3.59
Bases masked	36.7%	36.7%
Total interspersed repeats	33.9%	39%
Retroelements	30.2%	37.1%
SINEs	11.7%	8.3%
LINEs	11.4%	18.9%
LTR elements	7.2%	9.9%
DNA transposons	0.63%	0.45%
Unclassified	3.0%	1.5%

Gb, gigabase pairs; bp, base pairs; SINEs, short interspersed nuclear elements; LINEs, long interspersed nuclear elements; LTR, long terminal repeat.

The average amino acid similarity across gene blocks between *M. musculus* and *A. cahirinus* was 80%. Almost all forms of structural variations such as inversions, duplications, and insertions/deletions were detected in the synteny analysis between *A. cahirinus* and *M. musculus* (Supplementary Fig. 2). While this suggests dynamic structural changes to the rodent genome, the mutation rate analysis indicated a major peak at Ks ≪ 0.0, suggesting the majority of found gene pairs predate the *M. musculus–A. cahirinus* divergence (Supplementary Fig. 3).

The average nucleotide divergence between *A. cahirinus and M. musculus transcripts was 12%*. We estimated Ka/Ks for 33,197 mouse gene IDs that passed our alignment filtering. The vast majority of the genes had Ka/Ks values between 0 and 1 (mean 0.16, Supplementary Fig. 4), indicating purifying selection acting on protein-coding genes across rodents of the Muridae family. Of the rest, 38 significant gene IDs had both Ka/Ks > 1.0 and an adjusted *P*-value < 0.05; 34 of these contained DAVID IDs, many of which are predicted or known DNA chromatin/transcription (GO:0140110) and signal transducer regulation factors (GO:0060089, Panther GO-Slim molecular function). For example, 3 genes (*Dmrtc1b*, *Dmrtc1c1*, and *Dmrtc1c2*) were annotated by Uni-Prot and InterPro as being involved in the doublesex and mab-3 related transcription factor-like families, and 4 were enriched with the GO term meiotic cell cycle (GO:0051321), along with *Obox5* (oocyte specific homeobox 5) and *Rhox4* (reproductive homeobox 4C) transcription factors. Thirty-one of the significant genes with Ka/Ks > 1.0 were enriched with PANTHER GO-Slim terms for biological processes using the 33,197 aligned *M. musculus–A. cahirinus* genes as background. Enriched gene ontology terms included spermatid development (GO:0007286, 34.2-fold enrichment, FDR = 0.0184) and germ cell development (GO:0007281, 46.9-fold enrichment, FDR = 8.59E^−05^). These results suggest that important differences at the amino acid level between *M. musculus* and *A. cahirinus* contribute to post-speciation differences in reproductive development. This is a common result in comparative genomics analyses across mammalian species (Chai *et al.* 2021), and yet it may be indicative of *A. cahirinus*’ adaptations underlying their novel menstrual cycle, longer gestational times, and precocial births vs *M. musculus*’ more rapid estrous cycles and altricial birthing strategies (Bellofiore *et al.* 2017). Other *A. cahirinus* Ka/Ks > 1.0 genes with molecular function and biological process enriched terms of interest include catalytic activity (GO:0003824), metabolic processes (GO:0008152), and immune system processes (GO:0002376). For example, *Tcl1b3/4* (T cell leukemia/lymphoma 1B 3 and 4) is a protein serine/threonine kinase activator, and *Wfdc10* (WAP four-disulfide core domain 10) is an endopeptidase inhibitor with predicted roles in local immune responses in reproductive tissues based on mouse ENCODE RNA expression (Yue *et al.* 2014). *Gimap4* (GTPase of the immune associated nucleotide binding protein 5) regulates T lymphocyte activation and long-term survival (Limoges *et al.* 2021), *Ccnb3* (cyclin B3) is a known cell cycle and proliferation regulator, and *Slamf7* (signaling lymphocytic activation molecule family 7) is a signaling receptor that regulates innate and adaptive immune cell activation more broadly, which also underlies certain cancer progression in humans (Farhangnia *et al.* 2023). Thus, even our initial comparison between *A. cahirinus* and *M. musculus* protein coding level changes successfully revealed novel variants in key genes that regulate cellular processes. Understanding how these naturally selected protein coding changes differentially affect signal control and chromatin regulation mechanisms in *A. cahirinus* compared to other mammals will be of interest as this genome is investigated further to decode this animal's unique biology.

Out of 254,113 total genes across the 12 species, we assigned 247,445 (97.4%) genes to orthogroups, indicating that with our species sampling we were able to capture a high degree of orthologous gene relationships. In particular, we found that 97.1% of *A. cahirinus* genes were successfully assigned to orthogroups, indicative of the quality of our annotation. The highest percentage of genes assigned to an orthogroup was for guinea pig (98.9%), and the lowest percentage of genes assigned to an orthogroup was for opossum (94.5%), likely due to its being the only marsupial in our species sampling. We inferred the number of unique gene duplication events in the evolutionary history of *Acomys*, including 537 at the origin of therian mammals, 333 at the origin of eutherians, 4,061 for human, 828 for murine rodents, 2,493 for *M. musculus*, 2,246 for *A. russatus*, and 6,679 for *A. cahirinus*. We found 126 unique genes in 51 families unique to *A. cahirinus*. These results demonstrate that our *A. cahirinus* genome will be a useful tool in comparative genomics studies outside the context of pairwise comparisons to mouse.

To confirm the quality of our assembly and annotation as well as to identify a broad range of expressed transcripts, we performed RNA sequencing of several tissues. We aligned short-read RNA isolated from heart, liver, brain, and testis to the assembled genome. TPM for each annotated gene was calculated and used to determine expression levels among the 4 tissues for the 19,818 annotated genes. More genes with a TPM level > 2 were observed in the brain (11,450) and testis (11,938) compared to liver (7,593) and heart (5,194), and testis had the largest number of uniquely expressed transcripts (2,411) (Fig. 2b). This result is consistent with other studies that have demonstrated a high number of unique transcripts in brain in both *M. musculus* and *Rattus norvegicus* (Söllner *et al.* 2017) as well as a unique number of expressed transcripts in testis (Djureinovic *et al.* 2014; Uhlén *et al.* 2015). These results provide support for the high-quality of our genome assembly and demonstrate that tissue-specific expression analysis is feasible in order to better understand the regenerative capabilities of this species.

Diverse scientific disciplines have long studied *A. cahirinus* for their unique organismal and behavioral adaptations. Most recently, *A. cahirinus* have emerged as an exciting and experimentally tractable adult regenerative mammalian model, as their naturally selected capacity for antifibrotic scarless epidermal wound healing extends across multiple internal systems and different injury contexts. Hence, our highly contiguous, high-quality genome presented here will broadly benefit the growing *A. cahirinus* community and will accelerate more detailed investigations into the genetic and epigenetic mechanisms underlying *A. cahirinus*’ novel capacity to maintain organ regeneration as adult mammals.

## Supplementary Material

jkad177_Supplementary_Data

## Data Availability

The scaffolded genome assembly, RNA sequencing data, and original Nanopore data are available at NCBI under bioproject PRJNA935753. This Whole Genome Shotgun project has been deposited at DDBJ/ENA/GenBank under the accession JAULSH000000000. The version described in this paper is version JAULSH010000000. The scaffolded genome assembly and gff3 files are also available at https://doi.org/10.5281/zenodo.7761277. Additional annotation, alignment, and results from Ka/Ks analysis are available at https://doi.org/10.5281/zenodo.7734822. Orthofinder results are available at https://doi.org/10.6084/m9.figshare.23528349. Supplemental material available at G3 online.
