# Supplementary material for: Genome Report: chromosome-scale genome assembly of the African spiny mouse (Acomys cahirinus)
Source: G3 (Bethesda). 2023 Aug 8;13(10):jkad177. doi: 10.1093/g3journal/jkad177 (PMC10542272; doi:10.1093/g3journal/jkad177)
Supplement: jkad177_Supplementary_Data [file jkad177_supplementary_data.zip › G3-2023-404376_Supplemental_Material_Legends.docx]

# SUPPLEMENTAL MATERIAL

**Figure S1.** Read statistics after basecalling, generated with NanoPlot [(De Coster *et al.* 2018)](https://paperpile.com/c/JTXqpG/jW4Y).

**Figure S2.** Syntenic dot plot with Kn/Ks coloration.

**Figure S3.** Histogram of the log10-transformed synonymous mutation (Ks) values of the syntenic gene pairs found between *Mus* *musculus* (mm39) and *Acomys cahirinus*.

**Figure S4.** Distribution of Ka/Ks estimates for 33,197 protein-coding orthologs between *Acomys cahirinus* and *Mus musculus*.

**Table S1.** Summary of Oxford Nanopore sequencing statistics generated by NanoPlot [(De Coster *et al.* 2018)](https://paperpile.com/c/JTXqpG/jW4Y).

**Table S2.** Quast and BUSCO scores after each step of assembly, polishing, or scaffolding.

**File S1.** Concatenated output from KaKs_calculator2.0 analysis of mouse RefSeq transcripts.

**File S2.** TPM values for RNA sequencing data from heart, liver, brain, and testis.
