# Supplementary material for: Genome Report: chromosome-scale genome assembly of the African spiny mouse (Acomys cahirinus)
Source: G3 (Bethesda). 2023 Aug 8;13(10):jkad177. doi: 10.1093/g3journal/jkad177 (PMC10542272; doi:10.1093/g3journal/jkad177)
Supplement: jkad177_Supplementary_Data [file jkad177_supplementary_data.zip › G3-2023-404376_Table_S1.docx]

**Table S1.** Summary of Oxford Nanopore sequencing statistics generated by NanoPlot [(De Coster *et al.* 2018)](https://paperpile.com/c/JTXqpG/jW4Y).

| **Mean read length** | 20,158 | |
| --- | --- | --- |
| **Mean read quality** | 12.6 | |
| **Median read length** | 6,129 | |
| **Median read quality** | 12.6 | |
| **Number of reads** | 4,345,343 | |
| **Read length N50** | 62,867 | |
| **STDEV read length** | 32,666 | |
| **Total bases** | 87,593,195,281 | |
|  | | |
| **Quality cutoff** | **Number of reads (%)** | **Read length** |
| >Q5 | 4,345,343 (100.0%) | 87,593.2 Mb |
| >Q7 | 4,345,166 (100.0%) | 87,592.9 Mb |
| >Q10 | 3,444,710 (79.3%) | 74,862.6 Mb |
| >Q12 | 2,483,116 (57.1%) | 59,723.1 Mb |
| >Q15 | 930,916 (21.4%) | 23,692.5 Mb |
|  | | |
| **Top 5 longest reads** | **Mean base call quality score** | |
| 769,429 | 7.4 | |
| 730,505 | 7.3 | |
| 665,850 | 10.8 | |
| 661,387 | 11.2 | |
| 656,035 | 7.8 | |
