# Supplementary material for: Genome Report: chromosome-scale genome assembly of the African spiny mouse (Acomys cahirinus)
Source: G3 (Bethesda). 2023 Aug 8;13(10):jkad177. doi: 10.1093/g3journal/jkad177 (PMC10542272; doi:10.1093/g3journal/jkad177)
Supplement: jkad177_Supplementary_Data [file jkad177_supplementary_data.zip › G3-2023-404376_Table_S2.docx]

**Table S2.** Quast and BUSCO scores after each step of assembly, polishing, or scaffolding.

|  | **After purging duplicates** | **After polishing with medaka** | **After polishing with pilon** | **After scaffolding and annotation** |
| --- | --- | --- | --- | --- |
| **Quast statistics** | | | | |
| # contigs (≥ 0 bp) | 181 | 181 | 181 | 129 |
| # contigs (≥ 1000 bp) | 177 | 177 | 177 | 125 |
| # contigs (≥ 5000 bp) | 162 | 162 | 162 | 110 |
| # contigs (≥ 10000 bp) | 156 | 156 | 156 | 104 |
| # contigs (≥ 25000 bp) | 147 | 147 | 147 | 94 |
| # contigs (≥ 50000 bp) | 141 | 141 | 141 | 87 |
| Total length (≥ 0 bp) | 2,288,116,004 | 2,289,791,246 | 2,289,253,112 | 2,289,268,912 |
| Total length (≥ 1000 bp) | 2,288,113,194 | 2,289,788,406 | 2,289,250,289 | 2,289,266,089 |
| Total length (≥ 5000 bp) | 2,288,074,385 | 2,289,749,375 | 2,289,211,425 | 2,289,227,225 |
| Total length (≥ 10000 bp) | 2,288,033,368 | 2,289,707,944 | 2,289,170,056 | 2,289,185,856 |
| Total length (≥ 25000 bp) | 2,287,894,346 | 2,289,568,906 | 2,289,031,072 | 2,289,027,872 |
| Total length (≥ 50000 bp) | 2,287,707,297 | 2,289,381,919 | 2,288,844,248 | 2,288,790,854 |
| # contigs | 181 | 181 | 181 | 129 |
| Largest contig | 126,722,130 | 126,814,207 | 126,792,893 | 212,232,915 |
| Total length | 2,288,116,004 | 2,289,791,246 | 2,289,253,112 | 2,289,268,912 |
| GC (%) | 43 | 43 | 43 | 43 |
| N50 | 58,763,166 | 58,809,824 | 58,791,706 | 127,770,522 |
| N75 | 34,405,976 | 34,434,390 | 34,427,997 | 117,233,451 |
| L50 | 16 | 16 | 16 | 8 |
| L75 | 28 | 28 | 28 | 13 |
| # N's per 100 kbp | 0 | 0 | 0 | 1 |
| **BUSCO statistics** | | | | |
| Complete BUSCOs (C) | 3,290 (98.0%) | 3,300 (98.4%) | 3,305 (98.6%) | 3,304 (98.5%) |
| Complete and single-copy BUSCOs (S) | 3,218 (95.9%) | 3,225 (96.2%) | 3,232 (96.4%) | 3,233 (96.4%) |
| Complete and duplicated BUSCOs (D) | 72 (2.1%) | 75 (2.2%) | 73 (2.2%) | 71 (2.1%) |
| Fragmented BUSCOs (F) | 25 (0.7%) | 19 (0.6%) | 15 (0.4%) | 16 (0.5%) |
| Missing BUSCOs (M) | 39 (1.3%) | 35 (1.0%) | 34 (1.0%) | 34 (1.0%) |
