# Supplementary figures and images for: Genome Report: chromosome-scale genome assembly of the African spiny mouse (Acomys cahirinus)
Source: G3 (Bethesda). 2023 Aug 8;13(10):jkad177. doi: 10.1093/g3journal/jkad177 (PMC10542272; doi:10.1093/g3journal/jkad177)

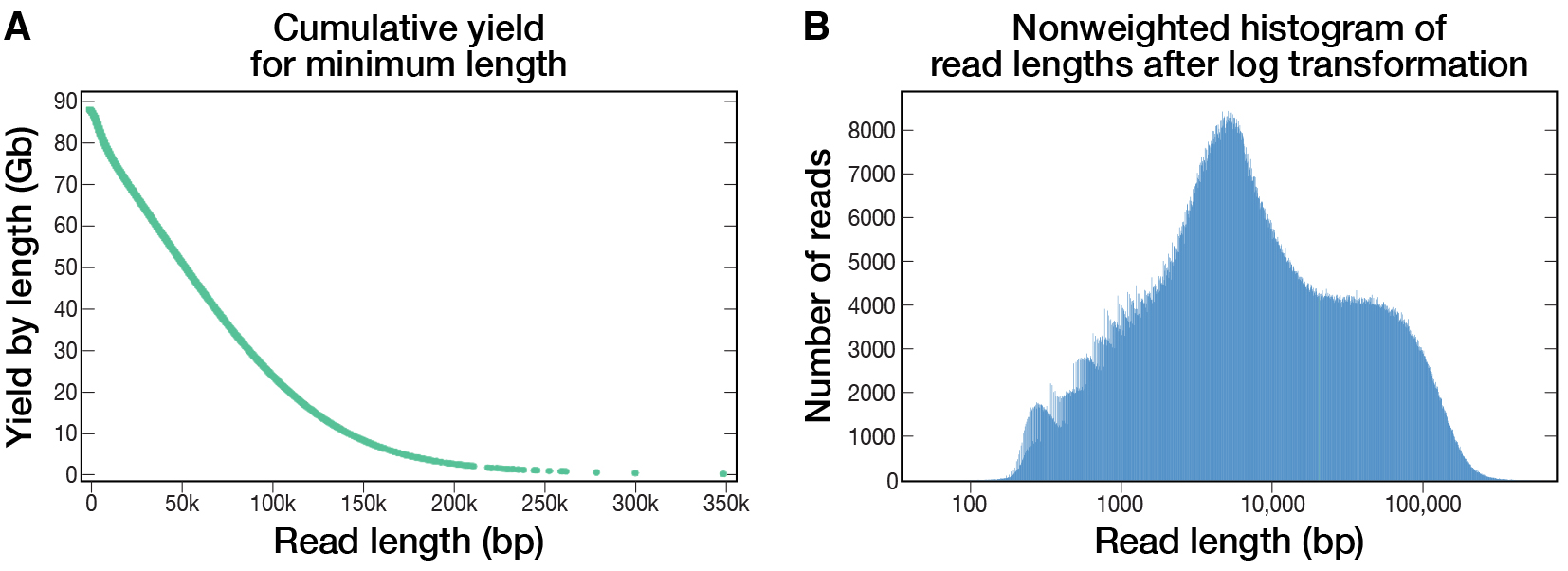

Supplement: jkad177_Supplementary_Data [file jkad177_supplementary_data.zip › G3-2023-404376_Figure_S1.jpg]

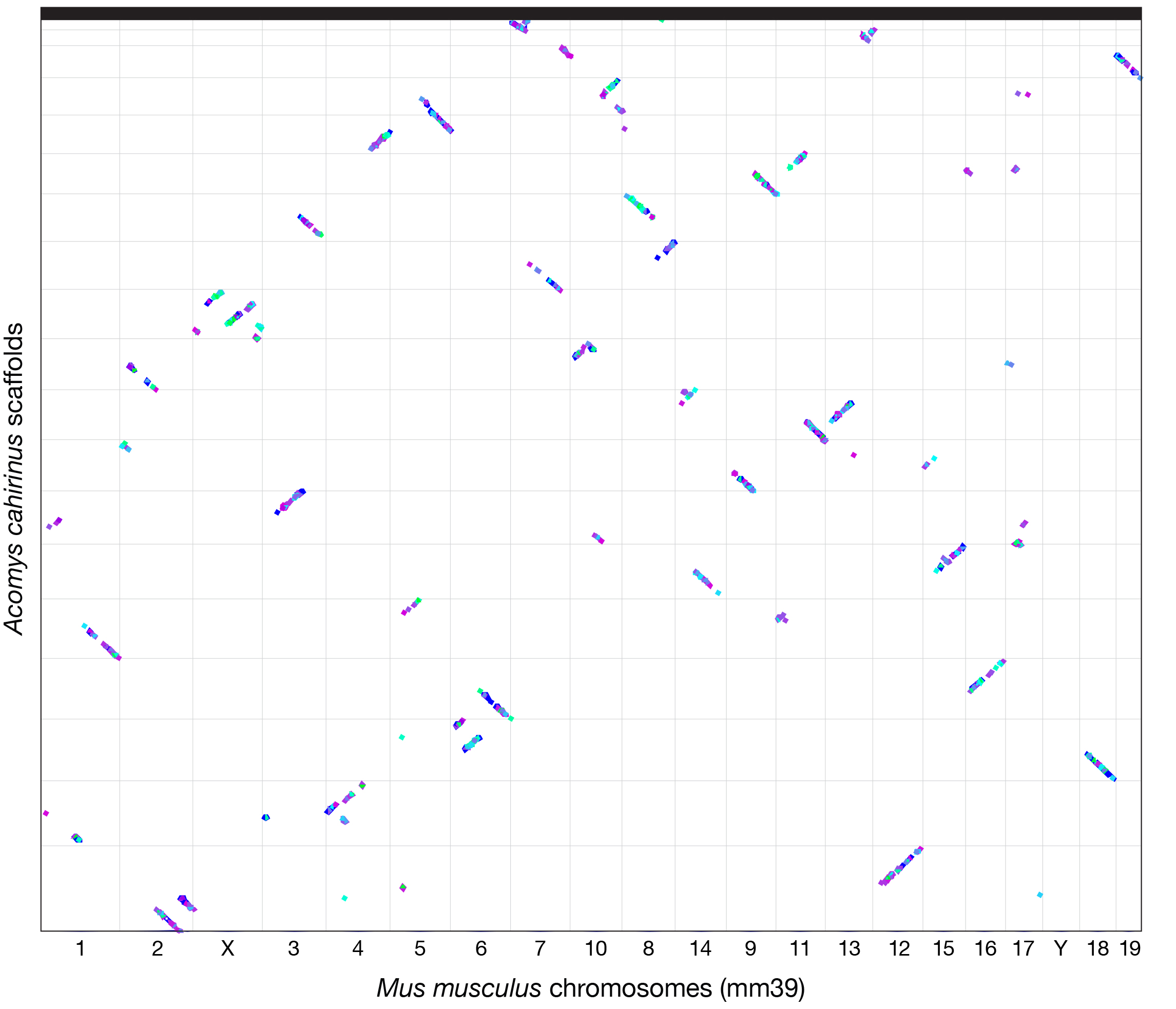

Supplement: jkad177_Supplementary_Data [file jkad177_supplementary_data.zip › G3-2023-404376_Figure_S2.jpg]

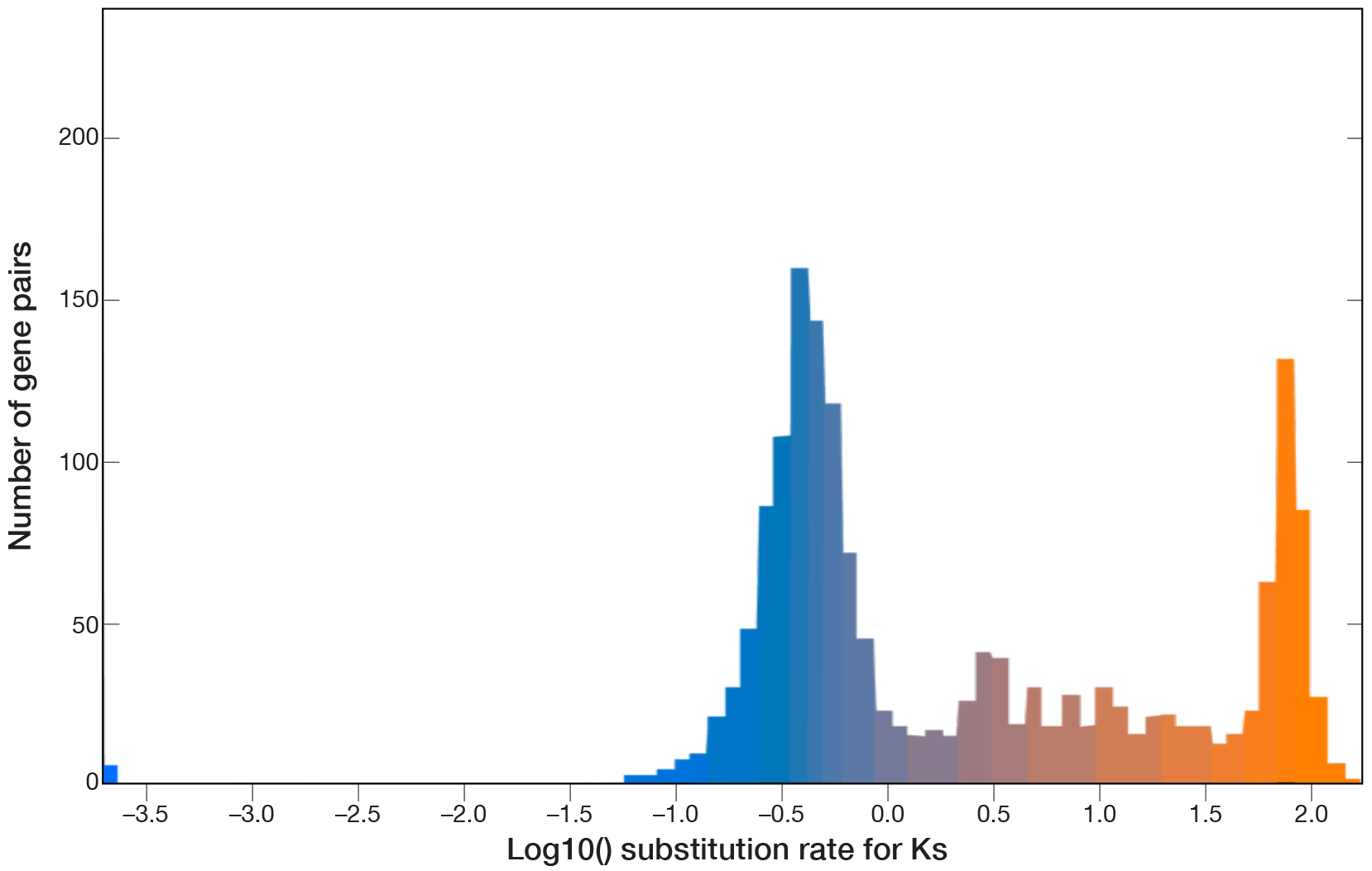

Supplement: jkad177_Supplementary_Data [file jkad177_supplementary_data.zip › G3-2023-404376_Figure_S3.jpg]

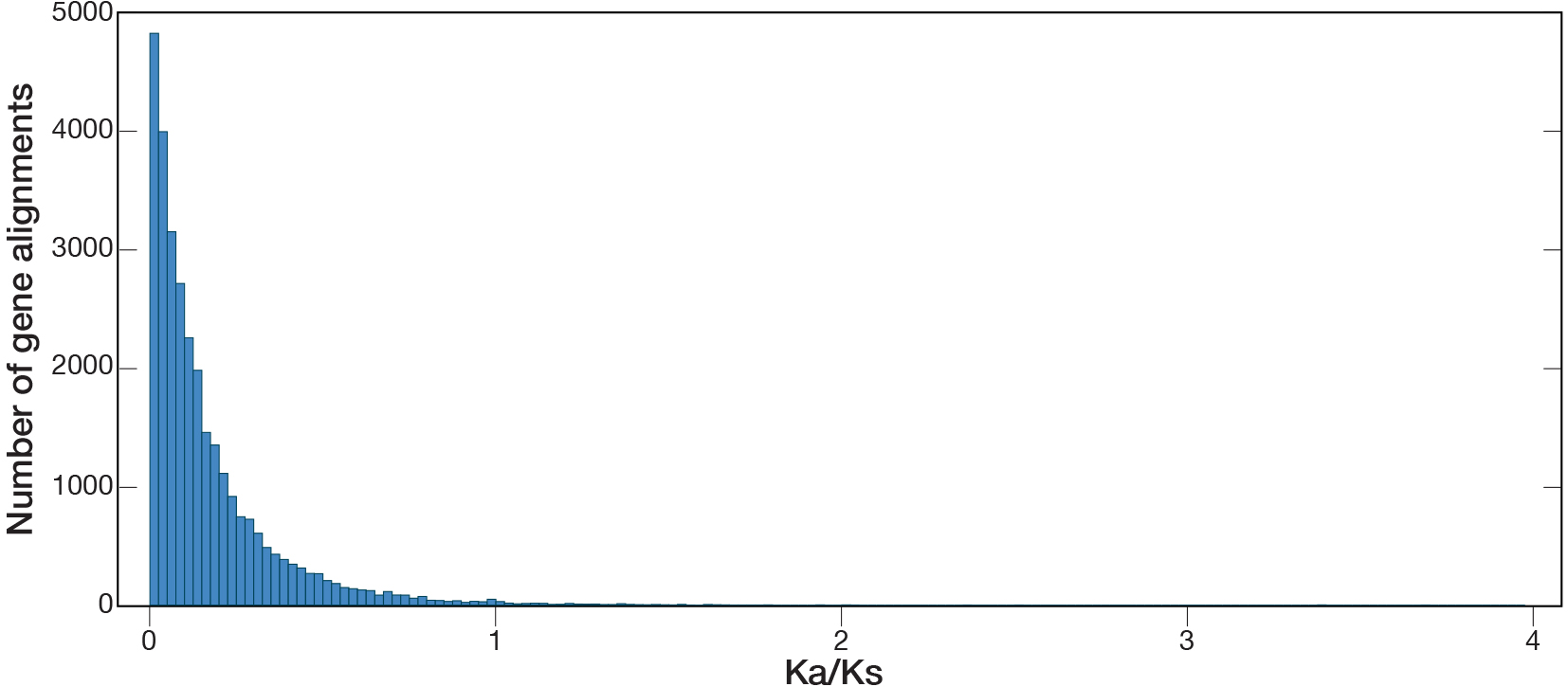

Supplement: jkad177_Supplementary_Data [file jkad177_supplementary_data.zip › G3-2023-404376_Figure_S4.jpg]
